# Process Mining of Football Event Data: A Novel Approach for Tactical Insights Into the Game

**DOI:** 10.3389/frai.2020.00047

**Published:** 2020-07-14

**Authors:** Pavlina Kröckel, Freimut Bodendorf

**Affiliations:** Institute of Information Systems, University of Erlangen-Nuremberg, Nuremberg, Germany

**Keywords:** football, soccer, process mining, sports analytics, tactics

## Abstract

The paper explores process mining and its usefulness for analyzing football event data. We work with professional event data provided by OPTA Sports from the European Championship in 2016. We analyze one game of a favorite team (England) against an underdog team (Iceland). The success of the underdog teams in the Euro 2016 was remarkable, and it is what made the event special. For this reason, it is interesting to compare the performance of a favorite and an underdog team by applying process mining. The goal is to show the options that these types of algorithms and visual analytics offer for the interpretation of event data in football and discuss how the gained insights can support decision makers not only in pre- and post-match analysis but also during live games as well. We show process mining techniques which can be used to gain team or individual player insights by considering the types of actions, the sequence of actions, and the order of player involvement in each sequence. Finally, we also demonstrate the detection of typical or unusual behavior by trace and sequence clustering.

## Introduction

Analyzing the tactical behavior in team sports is of paramount importance in sports performance analysis. The individual actions performed are of interest when analyzing the team's tactics. For quite some time, action frequencies of teams, and players have been the only way to gain insight into this performance aspect. However, this is not enough to get a complete picture of the performance, and especially the tactical behavior. Therefore, action/event sequences have been suggested for deeper insight into the game. One reason mentioned by Carling et al. (2008) is that “on-the-ball” activity, physical contact, and the sequence in which these actions occur contribute to physiological energy expenditure. This means that in addition to tactics, this kind of action analysis could give insight into player fatigue. Action sequences are chains of sequential single actions during a game (Schrapf et al., 2017). As the OPTA data are based entirely on event or action data, with timestamps and positional coordinates available, it is especially suitable for this type of analysis.

The paper presents a novel technique for sequence analysis of football event data and discusses its advantages and disadvantages for decision making in football. To the best of our knowledge, process mining has not been used for sports performance analysis until now.

## Process Mining

Process mining aims at discovering, monitoring, and improving real processes by extracting knowledge from event logs (van der Aalst, 2011). As a discipline, process mining sits between, on the one hand, machine learning and data mining, and on the other hand, process modeling and analysis (van der Aalst, 2011). Some of the answers which process mining can deliver are: (a) what really happened, (b) why did it happen, (c) what is likely to happen in the future, and (d) when and why do organizations and people deviate (van der Aalst, 2011).

There are different algorithms used in process mining, depending on the data available and the questions that need to be answered. Irrespective of this, process mining requires structured data, and specifically, event logs of business (or other) processes. The goal is to analyze event data from a process oriented perspective (van der Aalst, 2011). For a process mining algorithm to work, a few attributes must be available. These are “case ID,” “activity,” and “timestamp.” Other attributes in the dataset give additional information on the process and can be used as well in specific types of analysis in process mining, but they are not critical to the analysis.

There are three types of process mining: discovery, conformance checking, and enhancement (van der Aalst et al., 2012). The most often used type of process mining is discovery (van der Aalst et al., 2012). This technique converts an event log into a process model, without any a-priori information (van der Aalst, 2011). The discovered model can be in the form of a Petri net, BPMN, EPC, or UML activity diagram, but it can also be a social network model, depending on the perspective needed (van der Aalst et al., 2012). Conformance checking uses an event log and a model as inputs. It is used for finding discrepancies between the reality (event log) and the process model (van der Aalst, 2011; van der Aalst et al., 2012). The third type, enhancement, also uses an event log and a model as an input, but the information from the event log is used to improve the existing process model (van der Aalst et al., 2012). Finally, process mining may refer to different perspectives of the analyzed processes. These are explained below.

***Control-flow perspective***—ordering of activities. Here, the goal is to find a good characterization of all possible paths by deriving a process model that provides the best summary of the flow followed by most or all of the cases in the event log (ProM, 2017a). It can answer questions such as:

- Which tasks precede which other ones?
- Are there concurrent tasks?
- Are there loops?

There are several options for analyzing the case-flow. Some of the algorithms that can be used are the Alpha algorithm, the Heuristic Miner, Fuzzy Miner, and Inductive Visual Miner (IVM) (van der Aalst, 2016). A short comparison of these algorithms is presented in Table 1.

**Table 1:** Comparison of mining algorithms.

**Algorithm**	**Input**	**Output**	**When to use**
Alpha miner	Event log	Petri Net	Not recommended for real-life data.
Heuristic miner	Event log	Heuristic net	For real-life data with not too many different events.
Fuzzy miner	Event log	Fuzzy model	For complex and unstructured log data or for simplification of the model.
Inductive visual miner	Event log	Petri net or process tree	For discovering process delays, deviations, and animation of the model.

*Self-compiled based on Rozinat (2010), Leemans et al. (2014)*.

The answer of which algorithm should be used in a specific case is not a straightforward one. Table 1 provides a starting guideline when deciding which algorithm to use, but there are other options, and best is to test various algorithms and inspect the results. The *Alpha algorithm*, which was the first process mining algorithm developed, is not recommended for analysis of a real-world event log data (Rozinat, 2010). The *Heuristic Miner* was developed following the Alpha Miner to address its deficiencies and is therefore also able to simplify the process model by abstracting exceptional behavior and noise—by leaving out edges, i.e., connections between certain events (Rozinat, 2010). This algorithm is able to detect short loops and skipping of activities. However, it still shows rather complex process models (Buijs, 2017). The *Fuzzy Miner* interactively simplifies the process model by hiding some activities and paths, if desired (Rozinat, 2010). We use the *Inductive Miner* in our analysis because it was developed to overcome the disadvantages of other algorithms, and it shows a sound process model in the most user-friendly manner (Leemans et al., 2014).

***Organizational perspective***—focusing on information about the resources, which can be people, departments, roles, etc., and how they relate to each other. This relationship can also be represented as a social network based on the activities of the resources and can be used to find interaction patterns or evaluate the role of individuals (RapidProM, 2017). *Social network mining* is the most useful technique in the case of the organizational perspective, since network science is an area that studies interactions and relations between individuals. To derive sociograms from event logs, there are a few categories of metrics that have been developed (see Table 2).

**Table 2:** Types of social network metrics used for analyzing relationships from event logs.

**Metric category**	**Definition**	**Examples of metrics**
Metrics based on (possible) causality	Analyze how work moves among performers.	Handover of Work (HoW)
		Subcontracting
Metrics based on joint cases	Count how frequently two individuals are performing activities for the same case.	Working together
Metrics based on joint activities	Focus on the activities performed by individuals -> people are more similar if they perform the same activities.	Similar task metric
Metrics based on special event types	Consider the type of event.	Reassignment

*Self-compiled based on van der Aalst et al. (2005)*.

In this paper, we use two metrics for the analysis: the Handover of Work and the Working Together metrics, because the information is displayed in a similar way. Thus, we believe that by showing the results from these two metrics, the reader will understand what type of information can be extracted and how it is visualized.

The metrics based on (possible) causality consider how work moves among performers (van der Aalst et al., 2005). In a football game, it considers the flow of events between the players. For instance, there will be a *Handover of Work* between two players if there are two subsequent activities/events where the first is completed by player A and the second by player B. In addition to a direct succession, it is also possible to analyze “indirect succession using a “causality fall factor” β, i.e., if there are 3 activities in-between an activity completed by *i* and an activity completed by *j*, the causality fall factor is β^3^” (van der Aalst et al., 2005, p. 9). The subcontracting metric counts the number of times when player B executed an activity in between two activities done by player A. For instance, Player A -> Player B -> Player A. This could indicate that work was subcontracted from Player A to Player B.

Metrics based on joint cases ignore the causality and simply count how often individuals are performing activities within the same case, i.e., sequence of activities (van der Aalst and Song, 2004). Thus, the metric *Working Together* shows which players most often participate or “work together” in the same ball possession sequence. If two individuals often work together on cases, they are considered to have a stronger relation than individuals rarely working together (van der Aalst and Song, 2004; van der Aalst et al., 2005).

***Case perspective***—focusing on the properties of the cases. It can answer questions, such as (ProM, 2017a):

- What are the most frequent paths in the process?
- Are there any loop patterns in the process?
- What is the distribution of all cases over the different paths through the process?
- Can you select a subset of traces where specific paths were executed?
- Can you simplify the log by abstracting the most frequent paths?

Some options to answer the above questions with process mining are the *Trace Variants, Dotted Chart* visualizations, and the *Trace and Sequence Clustering*.

The basic idea of *Trace Clustering* is to split the event log into homogeneous subsets and for each subset to create a process model (Song et al., 2009). What this technique does is basically identification and clustering of similar sequences. The similarity is calculated based on a distance metric, usually the Euclidean or Hamming distance, while the clustering can be performed by using different algorithms, like k-Means or SOM (Veiga, 2009). A list of the algorithms available for clustering in ProM is presented in Supplementary Table 1.

Trace Clustering works by creating a set of profiles, each measuring a number of features for each case from a specific perspective (Song et al., 2009). In a second step, the distance between each case is measured by a distance metric; in this case, the Euclidean distance is used as it is found to be the most reliable. Finally, in a third step, similar cases are put together by using a clustering algorithm. Clusters can be analyzed independently from one another which improves the quality of the results for flexible environments (Song et al., 2009). Considering that football consists of 11 players who do not act according to a specific pre-defined process but rather based on quite a few distinct factors from their surrounding environment, one could reasonably assume that football can be considered a flexible environment within the process mining analytics area. Therefore, it would be interesting to see if and how trace/sequence clustering could be helpful for football performance analysis.

*Sequence Clustering* is based on a similar idea as Trace Clustering. However, this type of clustering is performed directly on the input data, i.e., no features are extracted from the sequences (Veiga, 2009). The plugin in ProM 5.7 has been implemented by Veiga (2009) whose algorithm is based on first-order Markov chains in which case the current state depends only on the previous state (Ferreira et al., 2007). The probability that an observed sequence is assigned to a given cluster is the probability that the observed sequence was produced by the Markov chain associated with that cluster, or simply the assignment of sequences to clusters is based on the probability of each cluster producing the given sequence (Ferreira et al., 2007; Veiga, 2009). Thus, a given sequence will be assigned to the cluster that is able to produce it with higher probability (Veiga, 2009). Veiga also adds two additional dummy states in the Markov chain: an input and an output state. This is necessary in order to represent the probability of a given event being the first or the last event in a sequence, which could be useful to distinguish between some types of sequences (Veiga, 2009).

In this paper, we use the SOM clustering algorithm and Markov chain clustering. SOM is used because it is very efficient with respect to computation time and is also quite robust concerning the results, especially for situations, where the characteristics of the process underlying an event log are largely unknown (Günther, 2009). The Markov chain clustering is preferred because it also discovers clusters without the analyst having to predefine the number of clusters. A detailed explanation of the algorithms is beyond the scope of this paper. For an overview of the SOM algorithm, the reader is referred to Si et al. (2003), and for more details on Markov chains, refer to Chung (1967).

***Time perspective***—analyzing the timing and frequency of events. If timestamps are available, it is possible to detect bottlenecks, monitor the utilization of resources, or predict the remaining processing time of running cases (van der Aalst, 2016). On its own, this perspective will most likely not be too interesting in a football scenario. However, combined with other perspectives, it can give interesting insights.

Each of these perspectives gives a different view of the process analyzed. The control-flow perspective relates to the “How” question, the organizational perspective to the “Who” question, while the case perspective answers the “What” question (ProM, 2017b). For a proper business understanding, users typically have to extract several models that describe different perspectives in the process analyses (Ingvaldsen and Gulla, 2008).

As seen, process mining is not a reporting, but an analysis tool, which is able to model and analyze complex processes (Rozinat and Gunther, 2015). Even though it works with historical data, it does not mean that it is limited to offline analysis, as the results can be applied to running cases (van der Aalst et al., 2012). Not all process mining types and perspectives can be applied to a football game scenario. From the three types of process mining mentioned, the discovery type is certainly applicable in this case, as conformance checking and enhancement require a model, which the discovered model from the OPTA log can be compared to. In football, there is no “perfect” or pre-defined model of the game process. Therefore, process mining can help with modeling the real-world process of what actually happened during the game. Finally, it is possible to view the event logs of the matches from all four perspectives discussed above.

## Analytics Approach and Tools

Each of the process mining analytic perspectives explained previously (case-flow, case, and organizational) is used in the analyses introduced here.

In a first step, the original OPTA data are pre-processed and converted into an event log data. This is followed by analysis of the resulting event logs, discovery of the process models, and interpretation. The potential of the applied analytics techniques to gain a tactical understanding in football is presented in the Discussion section.

One exemplary game is analyzed as the goal is to demonstrate the techniques. The game between England and Iceland is chosen because Iceland won, while England's team showed one of its worst performances in a tournament. Therefore, it is interesting to explore what process mining can reveal about the tactical player and team behaviors. A summary of the game is presented in Supplementary Table 2.

The tools used for the analyses are:

**ProM**— this is an open-source process mining software, which offers a wide range of algorithms and techniques to process and analyze event logs. There are also various plugins available to extend the analytics options further. In this paper, two versions of the software are used: ProM 5.2 and 6.7^1^ Some useful techniques such as the SOM trace clustering are missing in the later version, and therefore both versions are used in the analysis.
**Disco**— this is a proprietary process mining software developed by Fluxicon^2^ It is more user friendly than ProM and it is easier to read-in the event logs and get quick results. Although it has a better learning curve and results that are easy to interpret, it offers less analytical options than ProM. However, some of the techniques are easier for analysis, and therefore it is used in combination with ProM.

## Results

### Data Pre-processing

The first step of process mining is to pre-process the event log data from OPTA for the analyzed teams. Depending on the amount of additional information needed on each event, i.e., the attributes as described previously, the task can vary in complexity. The main issue, converting the log data into a format required by the process mining algorithms, is that each event in the OPTA log is described over several rows. Each row has different types and number of qualifiers which describe the event further. For instance, if a pass is analyzed, it can have qualifiers referring to the length of the pass, the angle, the x and y coordinates, etc. There are 36 qualifiers in total that can be used to describe the pass in more detail. Not all of them are used for each pass. The situation is similar with the rest of the 73 event types. Thus, it is a challenge to extract the relevant information in a way that the attributes of each event are added on a single row. A Python script tackles this challenge. Below, a few key steps executed by the code are introduced:

1. Eliminate unnecessary event types (formation change; deleted event, namely all events not related to ball possession or loss thereof)
2. Re-sort the data according to the scheme provided by OPTA, so that an accurate sequence of events can be obtained
3. Pivot the qualifiers (each tuple of qualifier ID and value is transposed to one column per qualifier)
4. Summarize data by event IDs (one row per event including all values for qualifiers)
5. Assign case IDs.

The output of the pre-processing step is a sequence of all events referring to the game with the ball. This means that a sequence for team A starts when the team gains ball possession and ends when the team loses the ball. An overview of the final data format is presented in Supplementary Figure 1. The minimum requirements for process mining are fulfilled by the columns “Seq. Num.”, “Event type,” and “Timestamp.” Additionally, the Period ID column (1—first half of the game), the x and y coordinates of the event in question, and its outcome (1—successful, 0—not successful) are available as attributes.

### Data Analytics

#### Case-Flow Perspective

**Figure 1:**
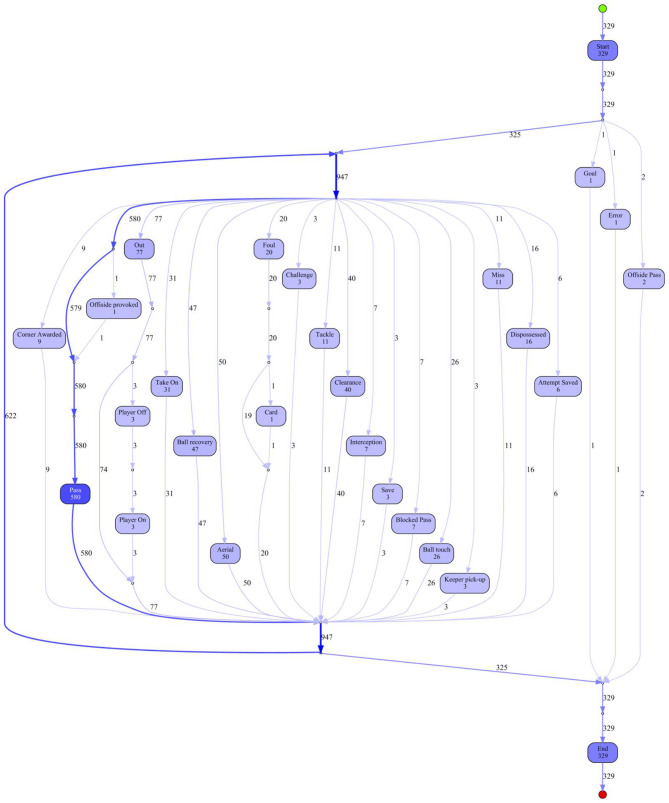
Process model of England's team by using the Inductive Visual Miner.

In a first step, the game is analyzed from a broader viewpoint, particularly the case-flow perspective. It gives a “helicopter” view of the sequences that happened for both teams and a summary of actions that characterize both the team and its players. As discussed, there are various algorithms that can be used to derive a process model from event log data. The algorithms are initially run with default settings, as this works well in most cases, at least in giving an initial idea of the usefulness of the algorithm in each individual case. In Figure 1 the results from the Inductive Visual Miner (IVM) are presented for the team of England. The IVM model for Iceland is presented in Supplementary Figure 2.

Both models display all activities and paths for each team during the game. The darker blue color indicates that those activities (events) occur more often during the game. Not surprisingly, the event “pass” is usually highlighted in this way. From an initial inspection of the models, we gain a first impression about the *event frequency*, e.g., for England it is immediately visible that the team had 580 passes or 9 corners awarded. But more interestingly, we can visualize the *dependency* between events, i.e., how often an event was followed by another. For instance, in England's team, once a “foul” (out of 20) was followed by a “card” event. Unfortunately, the model does not distinguish whether the foul was caused or suffered by England. Therefore, the process model for Iceland also shows that there are 20 fouls in the match.

The case-flow perspective does not seem to be very useful in a football case scenario as the frequencies of events are not interesting enough and are part of the traditional notational analysis.

#### Case Perspective

In a next step, various techniques and visualizations from the case perspective are applied.

**Figure 2:**
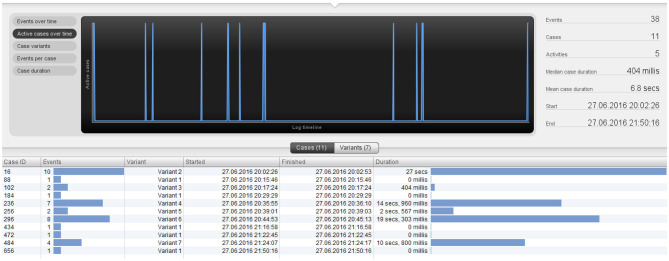
Instances ending in a “miss” event for England.

One option is to examine sequences that are of interest to the coach or his team. For instance, we can inspect the sequences that end with the event “*miss*” (any shot on goal which goes wide or over the goal). All the sequences, the time they occurred and duration, as well as players who started and ended the sequence can be inspected in this way. The overview of the sequences ending in the event “miss” for England are presented in Figure 2.

**Figure 3:**
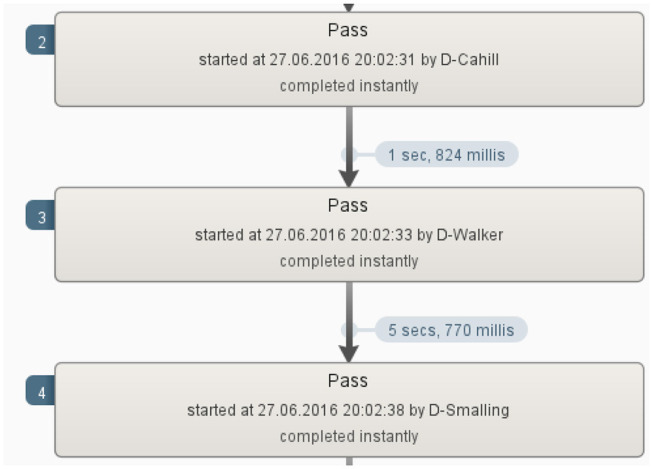
Closer inspection of a sequence ending in the event “miss”.

From Figure 2, we can see how many different variants these specific sequences have, i.e., how much they differ in terms of the type of events that precede a “miss” (in the case of England, there are 7 variants). Furthermore, we can examine how long it took between the starting event and the “miss” event (longest sequence lasts 27 sec). In 3 out of 11 instances, England missed a scoring chance after a longer passing sequence. Each sequence of interest can be individually inspected in order to investigate the exact order in which events happened as well as the player involved for each event. For instance, Figure 3 presents part of the longest variant type that lasts 27 seconds and happened once.

From Figure 3 we can see that the pass between Walker and Smalling took 5 seconds. Football coaches and experts can inspect in this way all sequences or parts of sequences that took the longest as well as who was involved and when. This will help them to strategize better but also understand what contributed to the success or failure of the team in that particular match.

The sequences also give more insights about the value of a player. When assigning credit to a player, standard statistics do not give enough credit to players who managed to keep the ball in possession by successfully getting out of tight situations (Gregory, 2017). One should not only look at players who shot toward the goal or made the key assist, as sometimes it can be much more difficult to enable that assist in the first place (Gregory, 2017). Process mining can give additional insights into a player's involvement in such situations. One option is to filter out all sequences that end in the following events: “attempt saved” and “goal”.

**Figure 4:**
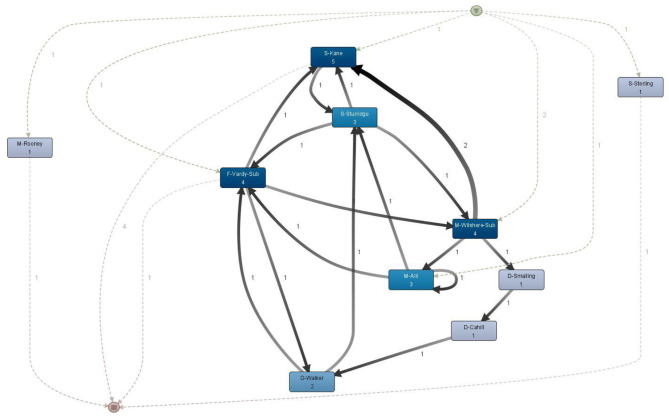
England's offensive sequences ending in a “goal” or “attempt saved”.

All sequences of England's team that end in one of the mentioned events are filtered out. The resulting process model is presented in Figure 4. Six sequences ended in “attempt saved”, while one sequence ended in “goal”. First, we can see which players were involved in these sequences, and which players started and ended the sequence. This is presented in Table 3. Based on Table 3C, Kane and Vardy are both frequent end-ers of offensive sequences in England's team. This makes these two players very valuable. However, we would like to know which players enabled these last key events, i.e., the shots on goal. Figure 4 shows that Rooney, Sterling, Kane, and Vardy are directly connected to the process endpoint—the red circle below. Thus, these players are process end-ers. Next, we can investigate which players are connected directly to the process end-ers and, thus, discover the players who enabled that final key pass (the shot on goal). Following the direction of the arrows in Figure 4, these players are Walker, Alli, and Sturridge (passing to Vardy) and Wilshere, Sturridge, and Vardy (passing to Kane). This leads to the conclusion that Sturridge is perhaps equally valuable as the sequence end-ers. The exact sequences can be closely inspected for details similarly to the information presented in Figure 3.

**Table 3:** Overview of players involved in offensive sequences.

**(A) List of all players involved in the offensive sequences**	**(B) Sequence initiators**	**(C) Sequence end-ers**
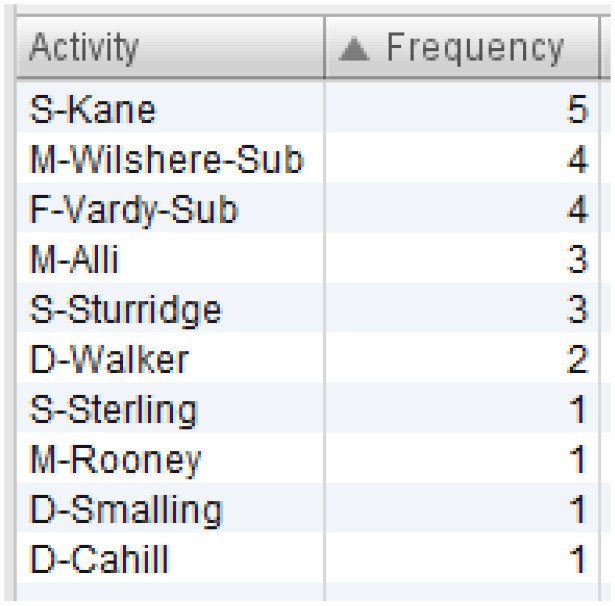	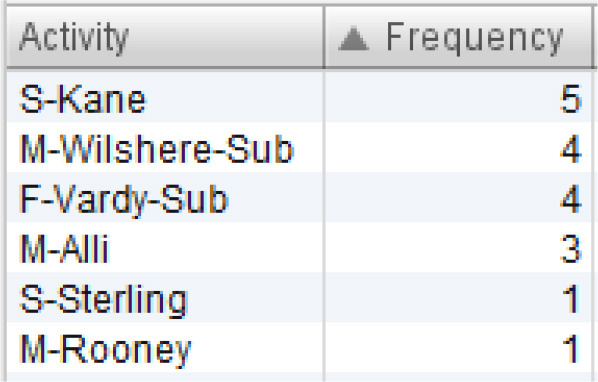	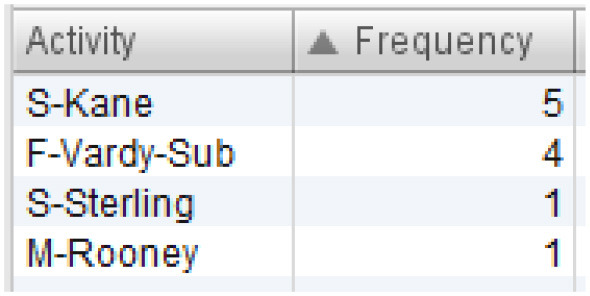

**Figure 5:**
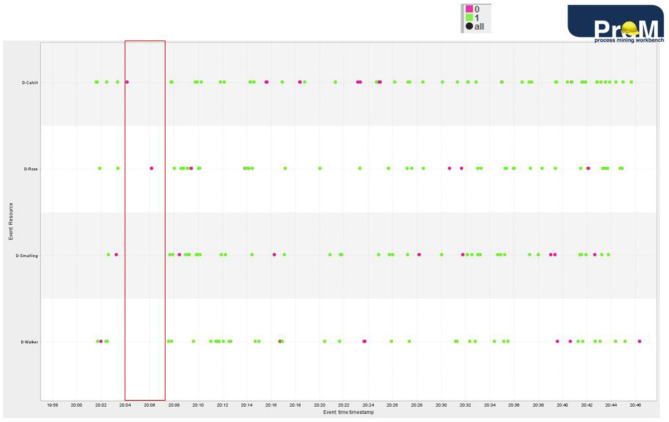
Outcome of activities of England's defenders in first-half.

The *Dotted Chart* is another visual analytics technique available in process mining. It is simple yet extremely useful for having a quick look at various aspects of the game and the players. It can be tweaked to present different dependencies between time, events, and players. One option is presented in Figure 5, but more are possible.

Figure 5 visualizes the outcome of events in which England's defenders were involved in the first half of the game. Red is unsuccessful outcome (for instance, ball lost) while green is successful outcome (for instance, successful pass). We can also see how often and when the defenders are engaged in the game. If we observe the timeframe between the first two goals, England's defenders show little action and the two visible actions in the highlighted part above have a negative outcome. We can also detect defenders who were involved in an unsuccessful activity, in this case Cahill and Rose.

As a final step in the case perspective analysis, we use trace and sequence clustering by means of SOM and Markov Chain algorithms.

To be able to apply SOM for trace clustering, we first need to build profiles of the traces based on some features. There are several options that can be chosen, and to do this right, one needs to ask what makes two sequences in football similar to each other. That would be the number and type of events in each sequence, the sequence duration, as well as the participants in each sequence, i.e., the players. We selected these as features based on which of the profiles of the sequences are built before the SOM clustering algorithm is used. There are several parameters for the SOM network which can be fine-tuned in the training process. These are briefly:

- Width and Height: this refers to the number of cells that should be used for the resulting rectangular grid. Each cell corresponds to one neuron.
- Radius: usually set to 2
- Random seed
- Training epochs.

**Figure 6:**
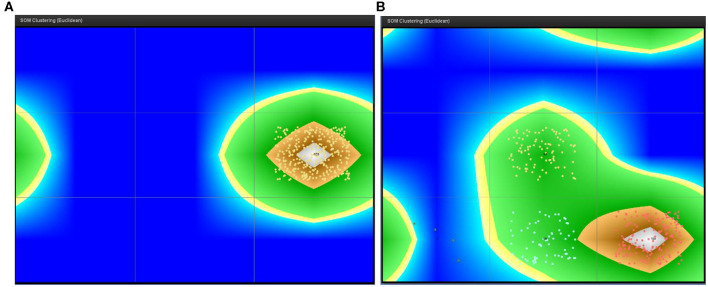
SOM trace clustering – England and Iceland. **(A)** England **(B)** Iceland.

In a few publications that use SOM for trace clustering (Günther, 2009; Song et al., 2009; Buddhika, 2016), parameter tuning is not discussed in detail. Usually, the Euclidean distance is used in combination with SOM and this combination is applied here as well. As to the width and height, there should not be more cells than there are traces (Günther, 2009). This is chosen usually intuitively after trial and error. The radius value which is used in step 5 of the SOM algorithm as well as the random seed parameters are usually kept at their default values of 2 and 999, respectively. This is the choice also for the analysis employed below. Additionally, the colors in the resulting map indicate the relationship between the neurons, i.e., neurons with a similar weight vector will be painted in a similar color (Günther, 2009). Clusters with many similarities, exhibiting normal behavior, are located in “high land” colored in green, while the clusters with exceptional cases are located at “sea” colored in blue (Buddhika, 2016). Finally, the cases in the same cell (the separate quadrants in Figure 6) belong to the same cluster (Song et al., 2009). These cells are calculated by using the U-matrix, a commonly used technique to cluster the SOM visually (Vesanto and Sulkava, 2002). A neuron n and the neurons in its Moore neighborhood N(n) on the output grid of the SOM represent points in the data space, while the sum of distances between n and the neurons in N(n) in the high-dimensional space is shown on a U-matrix as a height value (U-height) at neuron n (Lötsch and Ultsch, 2014, p. 249). The results of the SOM clustering are presented in Figure 6. Each dot represents one sequence (case), and all dots in the same cell belong to the same cluster.

The parameters are modified in a trial and error; however, the trace clustering for England's team results in a similar looking graphic as depicted in Figure 6A. There is no significant change in the outcome. On the other hand, for Iceland's team, the trial and error experiments result in more diverse clusters by changing the parameters' values. In the end, the result depicted in Figure 6B is chosen because it represents the average of the combination of results.

The trace clustering results for these two teams lead to the conclusion that England's players demonstrated a more homogenous behavior (all cases are in the same cell), while Iceland's players seem to be more creative (cases are split in four cells/clusters). This can be confirmed by popular opinions following the game.

The SOM results are confirmed by the sequence clustering and the generated Markov chains for recognized clusters. As we must first pre-define the number of clusters that need to be recognized, a trial and error for England's team reveals that when choosing a smaller number of pre-defined clusters (e.g., 2 to 4 clusters) the resulting clusters are of similar size and the Markov chains look relatively similar to each other. This again confirms the results from the SOM clustering that England's team plays in a rather predictable manner and their behavior is not exceptional or unique. The Markov chains though show more precisely a summarized overview of the main behavior of the team. In addition, we can see the probabilities that one event is followed by another. Finally, there are a few pre-processing steps that can be used for better clustering results, especially because without such pre-processing, the analysis can take more than 24 h. The options for pre-processing parameters and their values set for a football case scenario are presented in Table 4.

**Table 4:** Preprocessing parameters for Markov chain sequence clustering for England's team.

**Parameter**	**Value**
Min event occurrence (%)	3
Max event occurrence (%)	100
Min number of events in a sequence	2
Max number of events in a sequence	18
Min sequence occurrence	3
Max sequence occurrence	329

**Figure 7:**
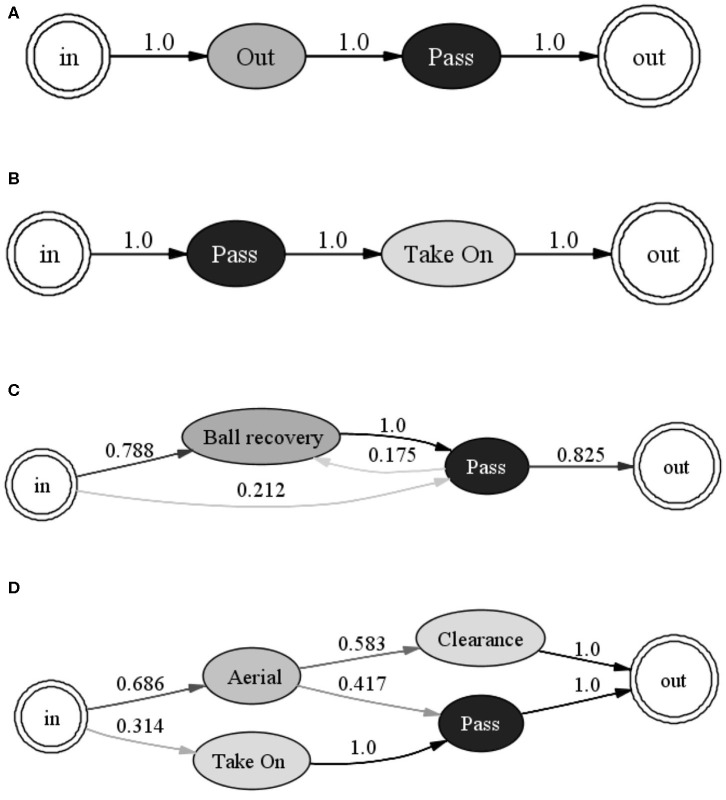
Markov chains for England's team. **(A)** Cluster 0: 30 instances, **(B)** Cluster 1: 3 instances, **(C)** Cluster 2: 33 instances, **(D)** Cluster 3: 35 instances.

We decided that an event should occur at a minimum 30 percent of all events and that there should be a minimum of 2 events in a sequence, to avoid rare and not interesting sequences of only one event. A sequence should also occur at least 3 times, while the maximum parameters are left at default. The number of clusters with these pre-processing steps applied is set to 4. The resulting Markov chains can be seen in Figures 7A–D.

This method gives an opportunity to easily drill down and get a quick overview of not only the events the players are mostly involved in and how often, but also the exact sequences that occur most often. We can also choose a higher percentage and check if there are some sequences that occur 50 or even 80 percent of the time. In England's case, when the minimum sequence occurrence is increased to 10 and the minimum event occurrence is increased to 40 percent, two clusters are generated with Markov chains in Figures 7A,D.

From England's Markov chains we can conclude that in roughly 30 percent of their game play, the ball is lost following just one pass after the ball was out of play. This means that they recover the ball and then lose it with just one pass (cluster 0). Furthermore, in 3 instances, England's team makes an unsuccessful dribble attempt past an opponent (cluster 1); there is a probability of 0.825 that they will lose the ball following a pass after a ball recovery (cluster 2), and finally, following an aerial duel, the probability for a clearance is 0.583 (cluster 3). This all speaks against England's team and shows at least some of the reasons behind their loss.

For Iceland's team it is more difficult to generate Markov chains that summarize the behavior well. One reason is that they are more resourceful than England's team. Thus, it is less likely that their play can be clustered in a meaningful way. Similarly to England, the minimum number parameters are modified while the maximum number parameters are kept at default. Using the same parameter setting for England, only 6 instances are left after the pre-processing steps. We get similar results by increasing the parameter “min event occurrence.” Therefore, after a trial and error we decided not to use the pre-processing parameters in Iceland's case and proceed with the clustering directly. The clustering results by pre-defining a different number of clusters are presented in Supplementary Table 3.

**Figure 8:**
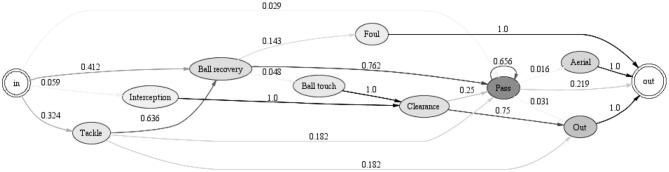
Markov chain for Iceland's team: cluster 3 with 34 instances.

The Markov chains and the instances are inspected for all the clusters in Supplementary Table 3. The 5 clusters summarize the behavior in the best way. For instance, cluster 3 is presented in Figure 8.

Looking at Figure 8, Iceland's team is often engaged in passing events (which is not that informative, as passes are the most frequent events for every team). However, Iceland has often events such as “ball recovery,” “interception,” “clearance,” and “out.” Furthermore, every time there is an interception, it is most likely followed by clearance, which is then followed by “out” with a probability of 0.75. This means that Iceland's team is quite successful in defending their half and intercepting the ball from the opponent's team.

**Figure 9:**
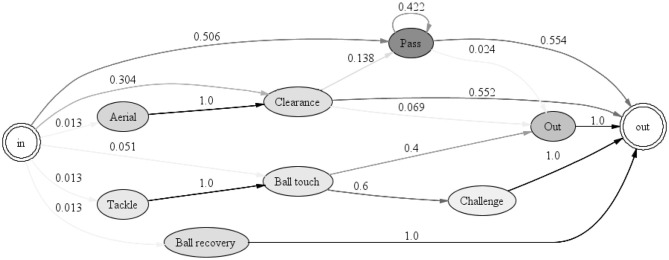
Markov chain for Iceland's team: cluster 1 with 79 instances.

Figure 9 shows that a “tackle” is most likely followed by a “ball touch” which in turn is followed by “out” (with a probability of 0.4) or “challenge” (with a probability of 0.6). This means that following a tackle, for Iceland's players the ball goes out of play for a throw-in or goal kick (out), or a player fails to win the ball as an opponent successfully dribbles past them (challenge). By using further analyses offered by process mining, for instance, the dotted chart (Figure 5), one can also check which players are involved in these unsuccessful events.

#### Social and Organizational Perspective of a Process

A process can be analyzed by looking at the organizational or social perspective. In the case of a football game, this mainly refers to viewing the process from the resource, i.e., the player perspective. As opposed to a typical social network analysis of a football game, where only passes are considered, this type of process mining takes into account all of the events available in the OPTA log.

**Figure 10:**
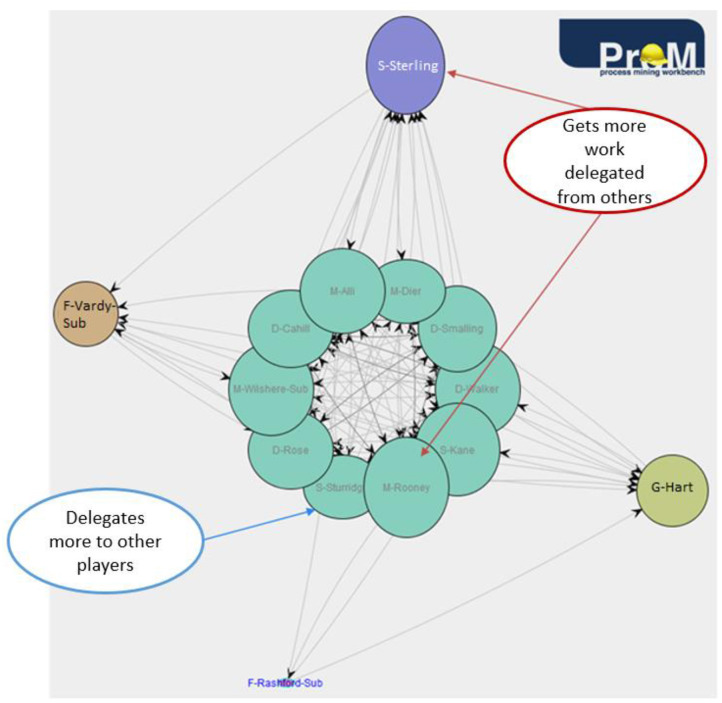
England–handover of work.

The first metric used in this analysis is the Handover-of-Work (HoW) metric. Figure 10 shows which players from England's team hand over work to other players in all action sequences. Only direct succession is considered. The HoW can be displayed by using different SNA metrics, like degree centrality, in and out degree centrality, betweenness, and closeness centrality. In this case, the degree centrality is chosen as it expresses the relation between the in and out degree of the connections between the nodes (ProM, 2017a). For Iceland's HoW graph, see Supplementary Figure 4. The graphs do not change significantly by using the other metrics.

In Figure 10, the colors add a cluster point of view to get a better visual perspective. The oval shape also has a meaning. The more vertically shaped nodes have a higher proportion of ingoing arcs, while the more horizontally shaped nodes have more outgoing arcs (ProM, 2017a). In this case, the clusters do not change significantly by removing more edges, which means that players from both teams have, on average, good participation over the course of the game, and display balanced participation. There are a few players that distinguish themselves from the others, however. In Iceland's team, Bjarnason, a midfield substitute player, has a distinctly vertical shape which means he gets more work delegated from the other players. Arnason, a defender, and Bodvarsson, a striker, also gets more work delegated than they themselves did for other players. In general, strikers would perhaps be players who are expected to have more incoming than outgoing arcs due to the nature of their position and, thus, the tasks that are required from them. Defenders, on the other hand, would ideally have more outgoing than incoming arcs. In England's team, Sterling and Rooney display slightly more vertical shapes, but overall, all players have a more balanced handover compared to Iceland's team.

The second metric investigated is the *Working Together* metric. This gives an insight into which two players often participate together in the same attacking sequence—for example, they pass the ball to each other in the same sequence.

**Figure 11:**
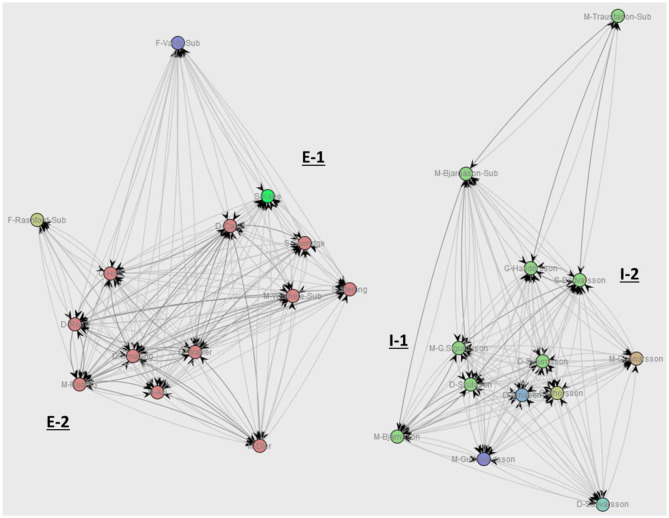
Working Together comparison between England **(Left)** and Iceland **(Right)**.

The network graphs in Figure 11 are generated by using the ISOM layout and degree centrality. Similarly to HoW, the graphs are not too different if other network metrics are used. This layout algorithm shows that in the team of England there are two more distinctive clusters of players that work together during attack and which consist of most of the players in the team: cluster E-1 consists of five players (S-Sterling, S-Kane, S-Sturridge, D-Cahill, M-Wilshere-Sub); cluster E-2 consists of six players (M-Rooney, M-Alli, D-Walker, D-Smalling, D-Rose, G-Hart). Three players from this team are isolated from the clusters: F-Vardy-Sub, F-Rashford-Sub, and M-Dier. The two substitute players come in minutes 60 and 86, respectively, so it is not surprising that they are outside of a cluster. Dier, on the other hand, plays as a central midfielder, and therefore has connections to both the E-1 and E-2 clusters. However, he is substituted at half-time by Wilshere, who did not perform well in an earlier match against Slovakia (Glendenning, 2016). From this SNA metric, Wilshere does appear to have stronger relationship with the players from the E-2 cluster as well. In Iceland's team, players are closely clustered together, with Traustason connected with the other substitute player, Bjarnason, the goalkeeper, and Bodvarsson. The midfielder, Bjarnasson, appears to have the closest connection to Skulasson and Sigurdsson. The defender Saevarsson works together occasionally with the rest of his teammates but does not seem to have a stronger relationship with a particular player. In the case of defenders, this behavior could also mean that the defender, by the nature of his task, more often interrupts a sequence of the opposite team. Skulasson works together with Bodvarsson quite often.

## Discussion

This paper presents an exploratory study to evaluate the potential and suitability of process mining for tactical football performance analysis. As seen, process mining is a collection of algorithms and analytics techniques which are widely used in other domains for analyzing all kinds of business processes. It has never been applied specifically to sports, however. Not all algorithms and visualization techniques of process mining are demonstrated in this paper and not all types of process mining can be used for performance analysis in football. The discovery type of process mining algorithms makes the most sense, as it can demonstrate the exact behavior of teams and players. The conformance checking type of process mining is, in our opinion, not useful in this scenario because one does not have the perfect process model according to which players need to behave during the game. Enhancement of the process model does not seem to be useful in this case either. However, the discovery algorithms and techniques prove to be very valuable for analyzing a football game from a process perspective. Table 5 presents a summary of the techniques and algorithms addressed in this paper.

**Table 5:** Process mining techniques and their insights in football.

**Perspective**	**Algorithm/analytics technique**	**Tactical insights into**	**Potential for decision support in football**
Case-flow perspective	Inductive visual miner	Team	Low
Case perspective	Filtering of specific sequences (e.g., offensive)	Team and player	Medium
	Instances inspector	Team and player	High
	Dotted chart	Team and player	High
	SOM trace clustering	Team	Medium
	Markov chain sequence clustering	Team	Medium
Social/organizational perspective	Handover of work	Player	High
	Working together	Player	High

The case-flow perspective with the various types of algorithms for discovering the process model has the lowest potential to improve decision making in football based on event data. We can only detect which event types occur most frequently and how events are connected. In addition, for some event types, this perspective is not useful. For instance, the event “foul” will appear in both teams, and it is not clear from the mined process model which team has made or suffered how many fouls. This is due to the way in which the sequences are extracted from the original dataset. As each event has different qualifiers, if all those are considered when creating the process model, there would be too many variants. As the idea of the model is to give a quick overview of what happened as well as some dependencies between the activities, such a level of detail is not focused on in this paper. Unfortunately, it is not possible to avoid this issue. However, this is the case only for a limited number of events. This type of visualization, as demonstrated by the Inductive Visual Miner, can usually be used to analyze the process from a time perspective, i.e., to check which activities last too long and discover bottlenecks. However, due to the nature of football, analyzing this process model from the time perspective would make less sense. We consider the time perspective as part of the other two perspectives (case and social perspectives) which are more useful for performance analysis in football.

The case perspective offers various useful visual analytic techniques and clustering algorithms which can give valuable insights into both the team and player behavior. For instance, once the process model has been generated with the Inductive Visual Miner, it is possible to drill down and filter out specific sequences, which are of interest for the decision maker. We filtered out the attacking sequences which gives answers to questions like:

- How many times did a team's action end up in events leading to shot-on-goal?
- Which events are those exactly? (e.g., miss, post, attempt saved, or goal)
- Which players were involved?
- When did these events occur and how long did the sequences last?

By using this option, it is possible to not only visualize the sequences leading to shot on goal for England's team but also to find out which players mostly started or ended a sequence. These analyses can be very useful in assessing the value of a player in a game.

Furthermore, clustering algorithms like SOM and first order Markov chains give a quick insight into the behavior of a team. Such analyses can be used, for example, during the half-time break in order to make tactical readjustments for the second half. Finally, social network analysis can be used for player insights. In this case, all event types occurring between the players are considered in the analysis in order to discover cooperation patterns between them. The two metrics that are applied, Handover of Work and Working Together, prove to be valuable in revealing important information about separate players. For instance, the Working Together metric can reveal which two players often cooperate in a sequence of ball possession, which in turn helps to plan tactical adjustments accordingly, especially concerning the defense of one own team. The Handover of Work metric can show which player is overwhelmed by having more work delegated from the other players. This could indicate fatigue or for the opponent can mean that that player should be the focus of their own defense.

We make an initial subjective evaluation of the potential usefulness of each technique presented in this paper for decision making in football. We base our evaluation on our knowledge of other data mining and visualization methods used in football performance analysis. However, this should be studied further by, for instance, conducting a study with coaches and other football experts, to gain an unbiased view on how useful such techniques are for actual decision making in football. Based on our initial results presented in this paper, process mining can be successfully used in addition to the traditional notational analysis for performance evaluation in football. It is an extension to the traditional analytics techniques that mostly consider the frequencies of actions. One major advantage is the possibility to visually infer patterns of interactions between the players and dependencies between different event types (goal, miss, duel, etc.), which has not been possible to achieve with other methods as, for instance, T-pattern analysis.

## Conclusion

Based on the presented results, process mining offers valuable techniques and algorithms, which give insights into players' and team's behavior. The results are usually quick and understandable. This type of analysis can be used for examining successful and unsuccessful sequence outcomes, establishing defensive strategies against specific players, and overall gaining tactical insights into team and player behaviors. It is more user friendly compared to methods like T-pattern analysis. The sequences of events are clearer. There are also various options for additional analyses of the sequences as well as filtering out and focusing on specific types of sequences, e.g., offensive or defensive, sequences ending in a specific event, or sequences in which a particular player is involved, sequences that last longest, etc.

Process mining offers even more possibilities for analyses of the action sequences. Therefore, future research can explore whether the conformance checking type of process mining would be helpful in a football scenario. For instance, it may be possible to use conformance checking techniques to simulate and test the outcomes of sequences by enhancing the event log with other events. Finally, the pitch zones based on the positional data could be integrated to check if they give even more detailed insights from the sequence data.

## Data Availability Statement

The datasets for this article are not publicly available because the data is proprietary data and subject to costs. Requests to access the datasets should be directed to the author.

## Author's Note

The paper is based on Chapter 8 of the first author's dissertation, which is also publicly available online (please refer to Kröckel, 2019).

## Author Contributions

PK wrote the paper and conducted the data analysis. FB revised the paper in terms of structure, content, writing style and grammar, acquired the data used in the analysis, and drafted the data analytics concept. All authors contributed to the article and approved the submitted version.

## Conflict of Interest

The authors declare that the research was conducted in the absence of any commercial or financial relationships that could be construed as a potential conflict of interest.

## Footnotes

^1^http://www.promtools.org/doku.php

^2^http://fluxicon.com/disco/
