# Supplementary material for: Process Mining of Football Event Data: A Novel Approach for Tactical Insights Into the Game
Source: Front Artif Intell. 2020 Jul 14;3:47. doi: 10.3389/frai.2020.00047 (PMC7861229; doi:10.3389/frai.2020.00047)
Supplement: Supplementary file 1 [file Data_Sheet_1.docx]

Supplementary Material

# Supplementary Figures


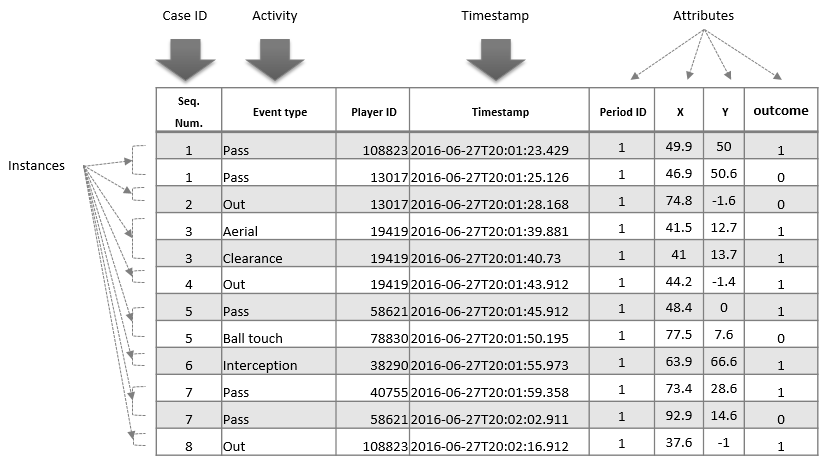


# Supplementary Figure 1. Pre-processed OPTA event log suitable for process mining tasks


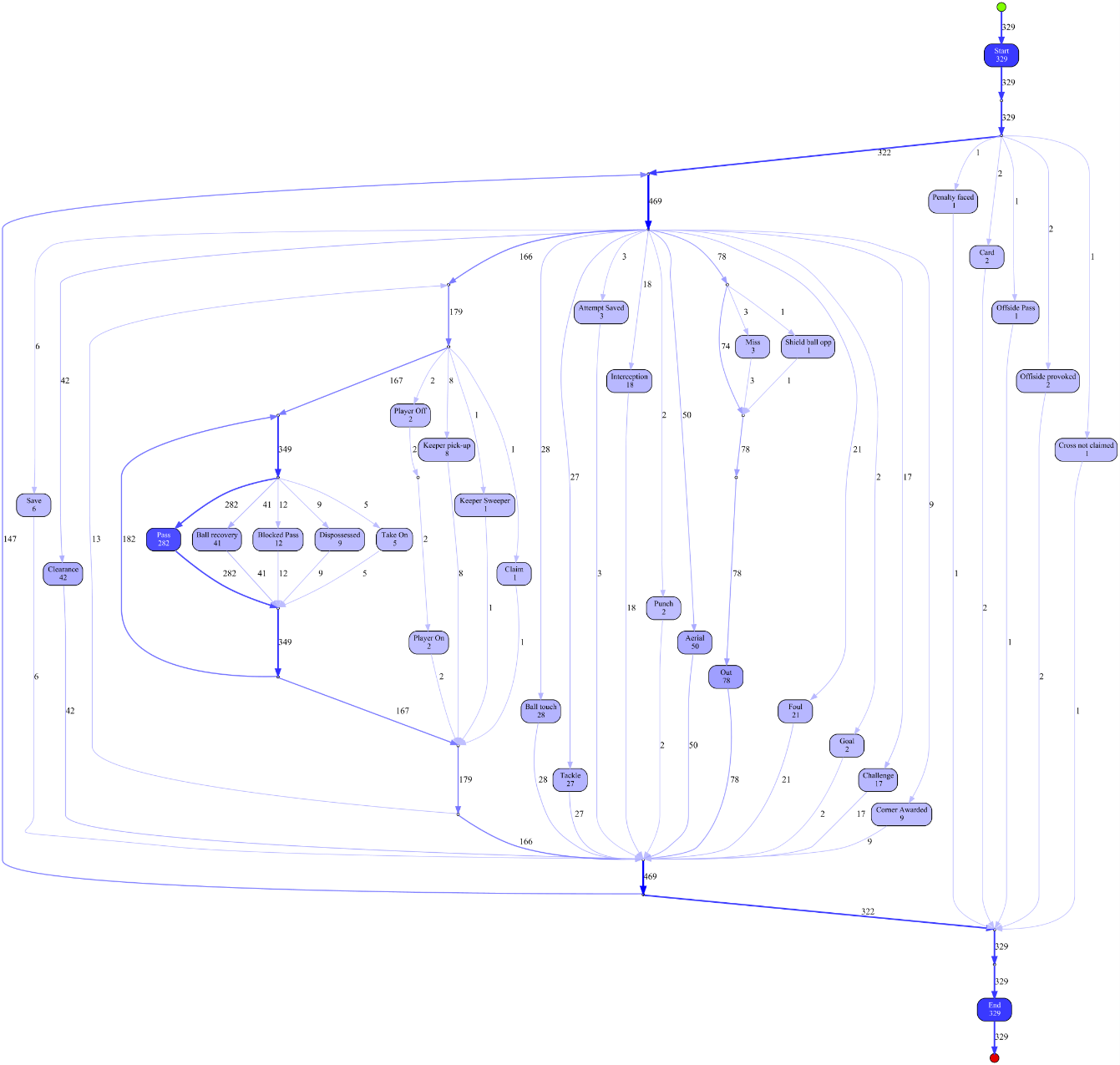


**Supplementary Figure 2.** Process model of Iceland’s team by using the Inductive Visual Miner


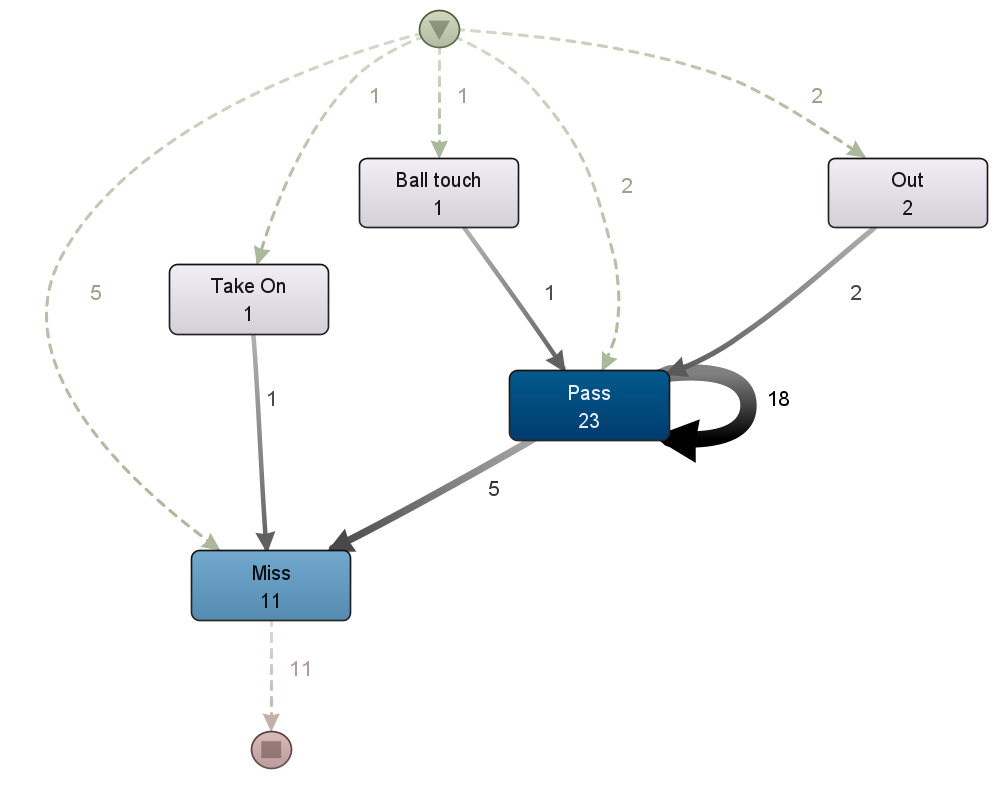


**Supplementary Figure 3.** Process model for sequences ending in “Miss” for England


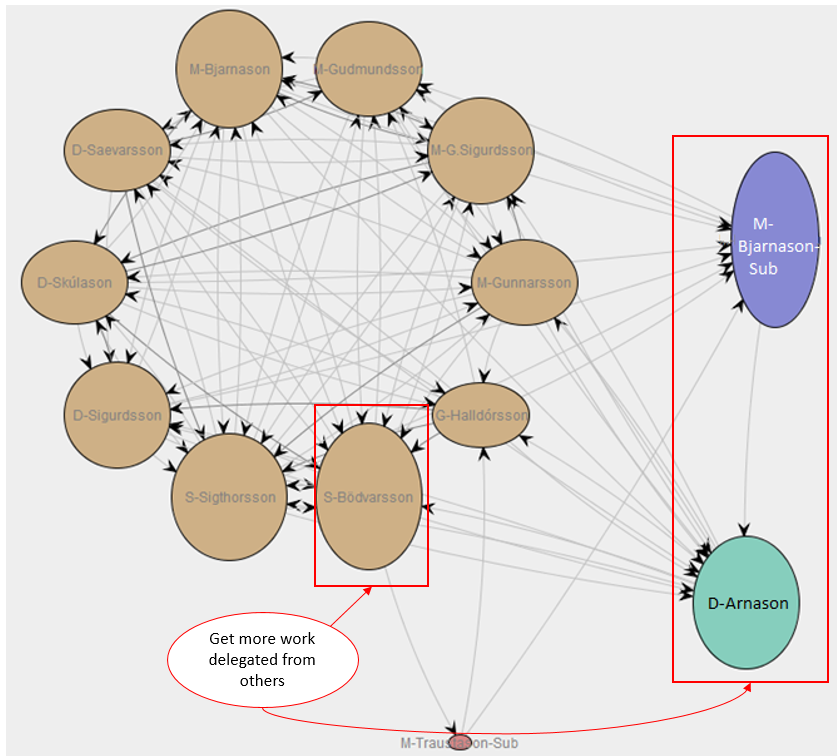


**Supplementary Figure 4.** Iceland – Handover of Work

# Supplementary Tables

Supplementary Table 1. Trace clustering algorithms in ProM

| **Algorithm** | **Description** |
| --- | --- |
| K-Means | The most commonly used in practice among partitioning methods, which constructs k clusters by dividing the data into k groups. |
| Quality Threshold Clustering | It is predictable (i.e., guaranteed to return the same set of clusters over multiple runs). |
| Agglomerative Hierarchical Clustering | Gradually generate clusters by merging nearest traces, i.e., smaller clusters are merged into large ones. |
| Self-Organizing Map | The aim of SOM is grouping similar cases close together in certain areas of the value range. |
| Markov clustering | Allows for identification of normal or exceptional behavior based on the input sequences. It discovers the clusters rather than requiring to set them beforehand. |

Source: Self-compiled based on Song et al., 2009;Hompes et al., 2015

Supplementary Table 2. Match statistics of England vs. Iceland’s game

| Date: June 27, 2016 | | |
| --- | --- | --- |
| England | **Performance indicators** | **Iceland** |
| 1 | Goals scored | 2 |
| 63 | Possession (%) | 37 |
| 18 | Total attempts | 8 |
| 5 | on target | 5 |
| 10 | off target | 3 |
| 3 | blocked | 0 |
| 0 | against woodwork | 0 |
| 7 | Corners | 2 |
| 2 | Offsides | 1 |
| 1 | Yellow cards | 2 |
| 0 | Red Cards | 0 |
| 6 | Fouls committed | 15 |
| 14 | Fouls suffered | 6 |
| 525 | Passes | 243 |
| 451 | completed | 173 |

Source: (UEFA, 2016)

Supplementary Table 3. Clusters and number of sequences for Iceland’s team

| **Number of clusters** | **Cluster** | **Number of sequences** |
| --- | --- | --- |
| 4 | 0 | 120 |
|  | 1 | 58 |
|  | 2 | 102 |
|  | 3 | 49 |
| 5 | 0 | 120 |
|  | 1 | 79 |
|  | 2 | 21 |
|  | 3 | 34 |
|  | 4 | 75 |
| 6 | 0 | 127 |
|  | 1 | 45 |
|  | 2 | 26 |
|  | 3 | 60 |
|  | 4 | 58 |
|  | 5 | 13 |
